# Nuclear envelope dysfunction and its contribution to the aging process

**DOI:** 10.1111/acel.13143

**Published:** 2020-04-15

**Authors:** Filipa Martins, Jéssica Sousa, Cátia D. Pereira, Odete A. B. da Cruz e Silva, Sandra Rebelo

**Affiliations:** ^1^ Neuroscience and Signaling Laboratory Institute of Biomedicine (iBiMED) Department of Medical Sciences University of Aveiro Aveiro Portugal; ^2^ The Discoveries CTR Aveiro Portugal

**Keywords:** chromatin organization, inner nuclear membrane, lamins, nuclear pore complex, nuclear transport, telomere maintenance

## Abstract

The nuclear envelope (NE) is the central organizing unit of the eukaryotic cell serving as a genome protective barrier and mechanotransduction interface between the cytoplasm and the nucleus. The NE is mainly composed of a nuclear lamina and a double membrane connected at specific points where the nuclear pore complexes (NPCs) form. Physiological aging might be generically defined as a functional decline across lifespan observed from the cellular to organismal level. Therefore, during aging and premature aging, several cellular alterations occur, including nuclear‐specific changes, particularly, altered nuclear transport, increased genomic instability induced by DNA damage, and telomere attrition. Here, we highlight and discuss proteins associated with nuclear transport dysfunction induced by aging, particularly nucleoporins, nuclear transport factors, and lamins. Moreover, changes in the structure of chromatin and consequent heterochromatin rearrangement upon aging are discussed. These alterations correlate with NE dysfunction, particularly lamins’ alterations. Finally, telomere attrition is addressed and correlated with altered levels of nuclear lamins and nuclear lamina‐associated proteins. Overall, the identification of molecular mechanisms underlying NE dysfunction, including upstream and downstream events, which have yet to be unraveled, will be determinant not only to our understanding of several pathologies, but as here discussed, in the aging process.

## Graphical abstract

Growing evidence has drawn attention to the nuclear envelope dysfunction as a hallmark of the aging process. Indeed, during aging and premature aging, several cellular alterations occur, including nuclear‐specific changes, particularly alterations in nuclear transport, chromatin organization, and telomere maintenance. Therefore, nuclear protein alterations are essential determinants of physiological and premature aging and may provide biomarkers for aging status and other pathologies like cancer.

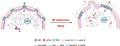

## 1 INTRODUCTION

In eukaryotic cells, the nuclear envelope (NE) is a protective barrier for the genome and a communication interface between the nucleus and the cytoplasm. This dynamic cellular compartment is mainly composed of three components: the nuclear lamina, a double membrane, and the nuclear pore complexes (NPCs). The double membrane comprises the inner nuclear membrane (INM) and the outer nuclear membrane (ONM), separated by the perinuclear space. The ONM is contiguous with the lumen of the endoplasmic reticulum (ER) (Dauer & Worman, 2009). These two membranes merge at numerous sites, giving rise to the NPCs. The latter are supramolecular structures that constitute channels for selective import and export of macromolecules as well as the diffusion of small molecules. Structurally, these protein complexes are constituted by the nucleoporins (Nups) that are crucial functional components of the diffusion barrier and transport channels (Knockenhauer & Schwartz, 2016; Terry & Wente, 2009). The ONM is encompassed by several specific integral membrane proteins that bind to the INM and cytoskeletal components, as well as by proteins found in the ER and ribosomes. In turn, the INM contains several transmembrane proteins that are in close association with both chromatin and the nuclear lamina. Nuclear lamina is a generally dense meshwork composed of A‐ and B‐type lamins and lamin‐associated proteins underlying the INM that provide structural, mechanical, and functional support to the nucleus (Burke & Stewart, 2013; Turgay et al., 2017; Worman, 2016). In mammals, A‐type lamins result from the *LMNA* gene alternative splicing, giving rise to both lamin A and lamin C isoforms. B‐type lamins result from expression of two distinct genes, namely *LMNB1* and *LMNB2,* originating lamin B1 and B2 isoforms, respectively. Interestingly, while the B‐type lamins form a looser network closely associated with the INM, the A‐type lamins’ network is more tightly spaced and remain in proximity to the INM facing the nucleoplasm (Delbarre et al., 2006; Goldberg, Huttenlauch, Hutchison, & Stick, 2008; Nmezi et al., 2019; Shimi et al., 2008, 2015; Xie et al., 2016). Another interesting aspect is that the lamins bind directly to chromatin via the lamina‐associated domains (LADs). Both A‐ and B‐type lamins bind to chromatin through interaction with INM proteins containing the LAP2–emerin–MAN1 (LEM) domain. Further, the lamin B receptor (LBR) that binds to B‐type lamins also interacts with heterochromatin protein (HP1) (Polioudaki et al., 2001; Ye & Worman, 1996) (discussed in section 3). Therefore, altogether the INM–nuclear lamina–chromatin association regulates several nuclear functions, including chromatin regulation, DNA replication and transcription, gene expression and cell signaling, as well as mechanotransduction, mitosis, and meiosis together with the ONM components (Wilson & Berk, 2010). Of note, although several lamin‐binding proteins have been identified it is possible that by resorting to recently developed highly reliable methods like APEX2, BioID, and 2C‐BioID (Chojnowski et al., 2018; James et al., 2019; Roux, Kim, Raida, & Burke, 2012), other interactors will be forthcoming.

Another important type of nucleocytoplasmic communication is the linker of the nucleoskeleton and cytoskeleton (LINC) complex that couples the nuclear interior to cytoskeletal structures through the building of communication bridges across the NE. The LINC complex is formed by the Sad1/UNC84 (SUN) proteins (INM proteins) and nesprins (ONM proteins) (Sosa, Kutay, & Schwartz, 2013; Starr & Fridolfsson, 2010).

The identification of mutations in the *LMNA* gene that causes premature aging disorders as Hutchinson–Gilford progeria (HGPS), mainly associated with defects and alterations in the nuclear proteins, increased the interest in the potential role of the nuclear lamina and nuclear lamina‐associated proteins as major regulators of the normal aging process. Premature aging disorders, or progerias, represent a powerful model for the study of potential mechanisms underlying physiological aging (Serebryannyy & Misteli, 2018). This hypothesis is strongly supported by several observations suggesting that sequestration of nucleoplasmic proteins at the nuclear periphery impacts cell stemness, the DNA damage response, changes in chromatin regulation, and telomere maintenance. Additionally, it has become evident that the loss of NE integrity leads to a gradual decrease in nucleocytoplasmic transport, selective loss and degradation of NE components, culminating in nuclear rupture, and aberrant transport of molecules between the nucleus and the cytoplasm (Robijns, Houthaeve, Braeckmans, & Vos, 2018). Together, these data indicate that NE integrity and its dynamic remodeling are pivotal requirements for cellular homeostasis and, consequently, to maintaining a healthy status. Therefore, when NE integrity is somehow perturbed, NE dysfunction and/or NE stress occurs, which appears to be a hallmark in several pathologies, like cancer and laminopathies, but also in viral infection and aging.

Physiological aging is mainly defined as a functional decline across lifespan observed from the cellular to organismal level (reviewed in López‐Otín, Blasco, Partridge, Serrano, & Kroemer, 2013). Therefore, the risk of developing complex diseases with aging increases. Importantly, the cellular function decline results from both intrinsic cellular modifications, including mitochondrial functional alterations and decreased differentiation potential, and also from environmental alterations, such as nutrient accessibility and endocrine signaling. Despite the extensive effort to determine the aging‐associated molecular and cellular changes, the precise underlying molecular mechanisms remain elusive. Nonetheless, there are several well‐recognized cellular and molecular hallmarks of aging, such as changes in nutrient availability, intercellular signaling, mitochondrial functions, imbalanced proteostasis, and cellular senescence. These are accompanied by nuclear‐specific alterations, such as enhanced genomic damage and instability and telomere erosion (reviewed in López‐Otín et al., 2013).

Here, we review and discuss the contribution of NE dysfunction to aging, with particular focus on alterations in the NPCs and nuclear transport as well as on changes in the nuclear lamina and its associated proteins, which are responsible for chromatin regulation and telomere maintenance.

## 2 NUCLEAR TRANSPORT

NE dysfunction is generated as a consequence of nuclear periphery integrity loss leading to gradual decrease in nucleocytoplasmic molecules transportation and selective deterioration of NE components, culminating in NE rupture, and aberrant nuclear transport (reviewed in Robijns et al., 2018). Remarkably, these alterations might have additional impacts on genome instability, chromatin remodeling, and gene expression (discussed in sections 3 and 4).

The NE remodeling is a dynamic and restricted process of cells with proliferative capacity. Hence, in proliferating cells, during interphase, the nuclear genomic content doubles and the NE surface area expands considerably, but also NPCs and other NE components are newly synthetized and integrated into the NE membranes (Webster, Witkin, & Cohen‐Fix, 2009). NE components have specific limited lifespan, being degraded when their life cycle ends and when damaged. The NPCs consist of an extremely complex macromolecular structure composed mainly by Nups that are associated with stable sub‐complexes, such as the scaffold complexes (Nup107‐160 complex and Nup93‐205 complex), the peripheral components (Nup214 complex, Nup98 complex, Nup62 complex, Nup50, Nup153, and Tpr), and three transmembrane proteins responsible for anchoring the NPC to the NE, named Ndc1, Pom121, and Gp210 (D’Angelo & Hetzer, 2008). Most of the peripheral nucleoporins comprise phenylalanine‐glycine‐rich repeats (being called FG‐Nups; e.g., Nup153), through which they interact with nuclear transport receptors providing a selective barrier for the diffusion of molecules larger than 60 kDa (Rabut, Lénárt, & Ellenberg, 2004). Of note, while peripheral Nups have a high turnover rate, scaffold Nups are only disassembled and reassembled during mitosis. This indicates that the scaffold complex is constant during interphase and remodeled throughout the mitotic phase of cell cycle (Daigle et al., 2001; Rabut et al., 2004). In nonproliferative cells, NPC scaffold members present a long‐life residence at the NPC with no turnover. This suggests that the NPCs and their functional performance declines across lifespan and may cause nuclear dysfunction and, consequently, loss of nuclear integrity.

**FIGURE 1:**
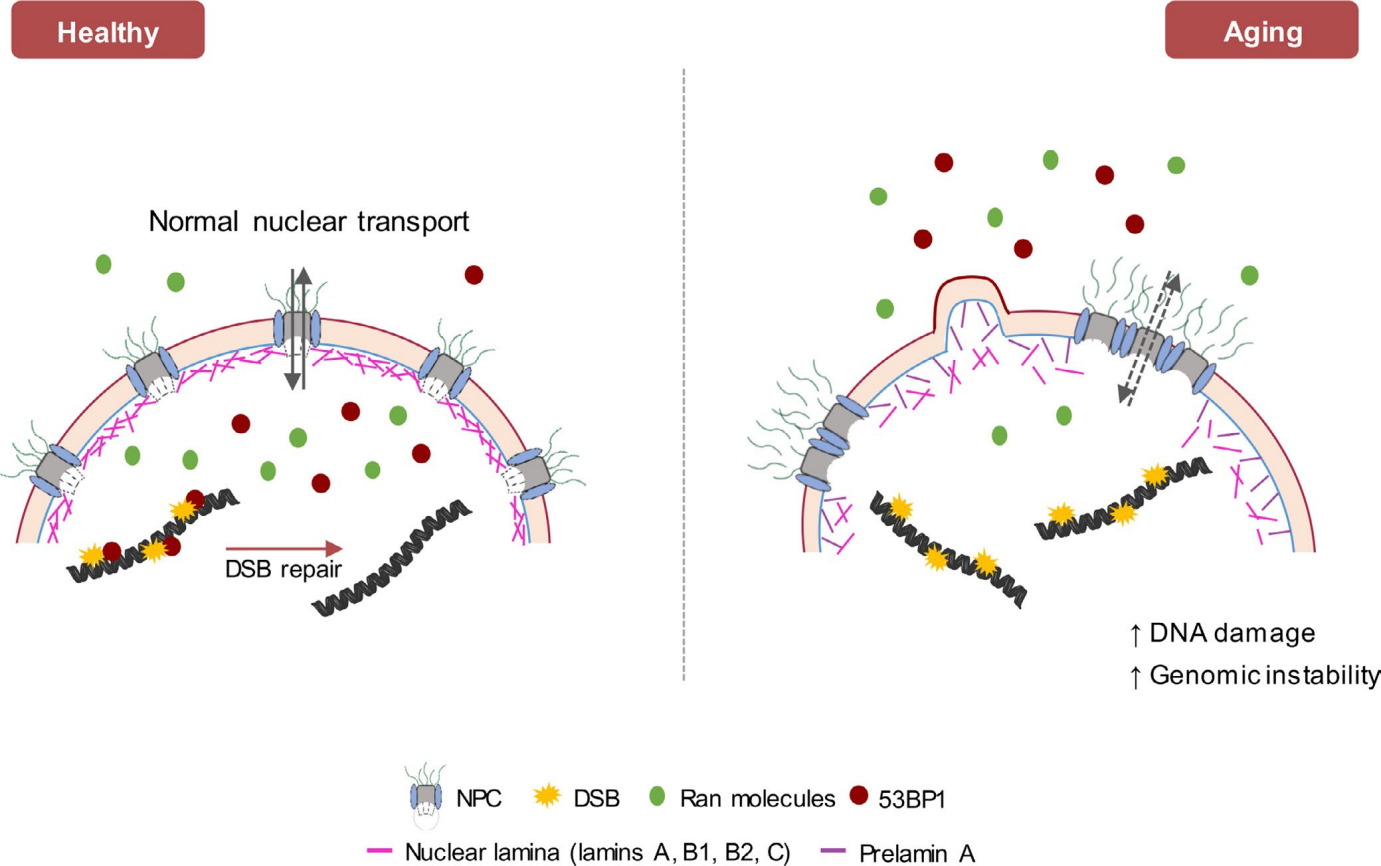
Mechanisms responsible for nuclear envelope dysfunction during aging. With age, prelamin A accumulates and induces NE invaginations, trapping Nup153. This results in disruption of the nuclear basket of the NPC, leading to NPC clustering and degradation that decreases NE selectivity. The mislocalization of Nup153 affects the Ran gradient by interfering with its import into the nucleus, which in turn reduce the nuclear import of large molecules, namely 53BP1. 53BP1 is a key protein for DSB repair and the decrease of its import to the nucleus compromises the DNA damage repair, leading to the accumulation of DNA damage in the cells. DSB, DNA double‐stranded break; NE, nuclear envelope; NPC, nuclear pore complex

**FIGURE 2:**
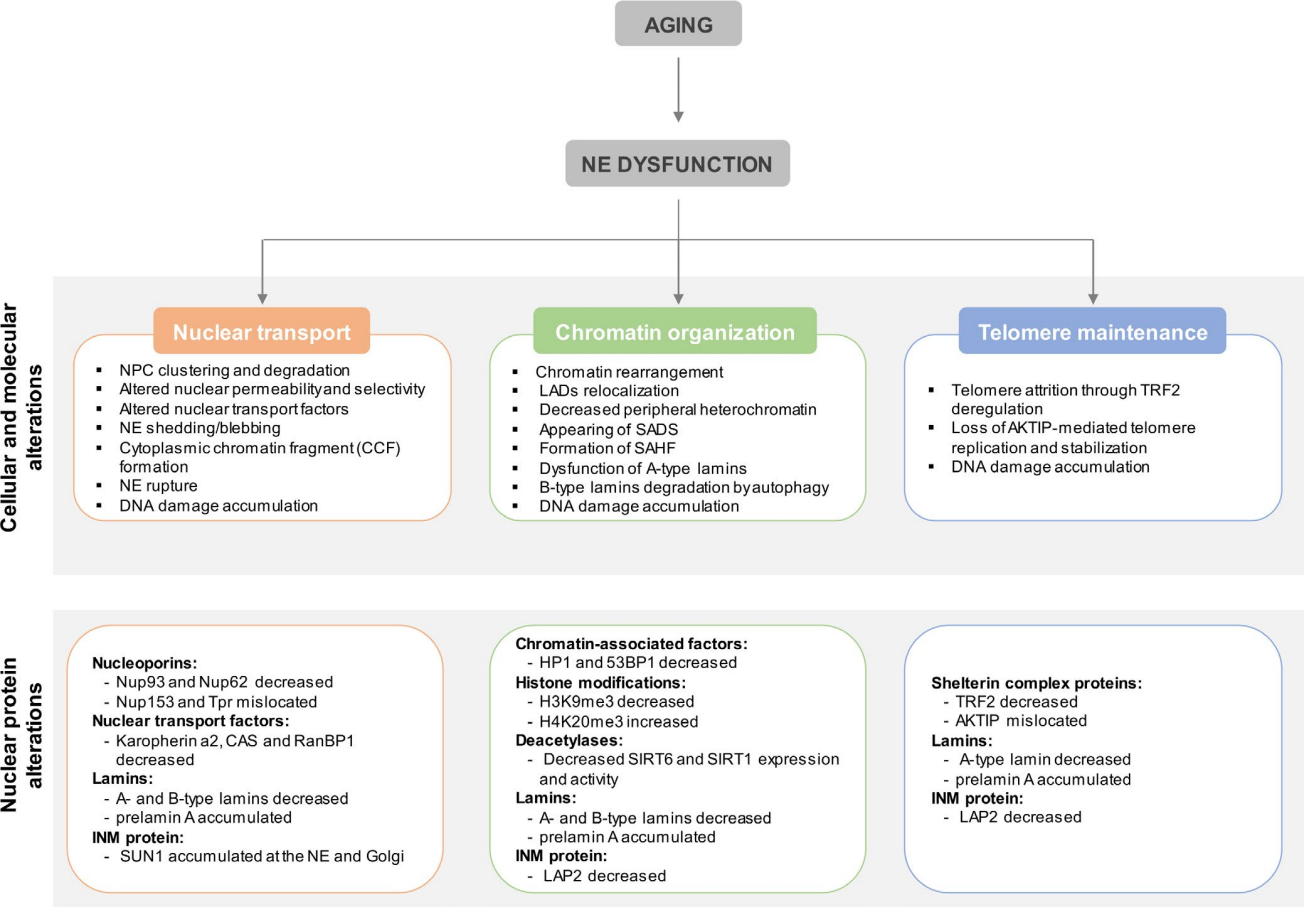
Summary of the contributions of nuclear envelope dysfunction to aging. Nuclear transport, chromatin regulation and telomere maintenance changes upon NE dysfunction in physiological and premature aging. HP1, heterochromatin protein 1; LADs, lamina‐associated domains; NPC, nuclear pore complexes; SADS, senescence‐associated distention of satellites; SAHF, senescence‐associated heterochromatin foci

Recently, Robijns et al. (2018) proposed three forms of NE stress that might affect the NE transport: NE erosion, NE shedding/blebbing, and NE rupture generated by defects in NPCs and/or other NE constituents, leading to NE dysfunction. NE erosion results from NPC clustering and degradation that will significantly decrease NE selectivity. In aged vascular smooth muscle cells (VSMCs), prelamin A, the lamin A precursor protein, accumulates inducing NE invaginations, trapping Nup153, which consequently compromises the Ras‐related nuclear protein (Ran) gradient, and large cargo transportation to the nucleus. As a result, 53BP1 is not efficiently recruited to DNA damage sites; thus, damage increases (Salamat, Dhar, Neagu, & Lyon, 2010; Warren & Shanahan, 2011) (Figure 1 and Table 1). Briefly, it was established that Nup153 is essential for Ran nuclear localization, and when the Ran gradient is perturbed, there is a commitment of 53BP1 nuclear import, consequently inducing genome instability (Cobb et al., 2016), potentially by propagating DNA damage (Figures 1, 2 and Table 1).

**TABLE 1:** Nuclear envelope proteins associated with or altered in the aging process

NE protein	Aging model	Alterations observed with aging	Cellular and molecular alterations	References
Lamin A/C	Physiological aging models: Human fibroblasts extensively passaged in culture Human centenarian fibroblasts Human aged‐iPSCs Human VSMCs from old donors Human aged‐iPSCs	Prelamin A accumulation at the nuclear periphery Depletion of nucleoplasmic lamin A/C Increase of lamin A expression in human aged‐iPSCs	Prelamin A/progerin accumulation cause the disruption of lamin A/C‐related functions, culminating in alterations in the nuclear structure and functions	Lattanzi et al. (2014), Petrini et al. (2017), Ragnauth et al. (2010), Scaffidi and Misteli (2006)
	Premature aging models: Human HGPS fibroblasts Human HGPS coronary arteries Zmpste24‐deficient mouse MEFs	Accumulation of progerin in the nuclear membrane in a cellular age‐dependent manner Depletion of nucleoplasmic lamin A/C induced by progerin expression		Candelario, Sudhakar, Navarro, Reddy, and Comai (2008), Goldman et al. (2004), Liu et al. (2005), McClintock et al. (2007), Olive et al. (2010), Rodriguez, Coppedè, Sagelius, and Eriksson (2009), Scaffidi and Misteli (2005), Vidak et al. (2015)
Lamin B1	Human diploid fibroblasts (WI‐38 cell line) extensively passaged in culture Human fibroblasts extensively passaged in culture Human fibroblasts from old donors Human progeria fibroblasts (mutation E145K) Human HGPS fibroblasts Senescent human fibroblasts Human aged‐iPSCs	Reduction of lamin B1 expression in human senescent and progeria fibroblasts, and in aged‐iPSCs Depletion of lamin B1 from the perinuclear region	Lamin B1 reduction is a consequence of senescence caused by activation of p53 and Rb Lamin B1 reduction in senescent cells is associated with unselective permeability of the NE Nuclear‐to‐cytoplasm chromatin blebbing (CCFs formation) in senescent cells is associated with the lamin B1 reduction Lamin B1 reduction seems to have a role in distension of satellite DNA, contributing to the appearance of SADs Lamin B1 protein turnover is achieved by autophagy in senescent cells Lamin B1 mRNA decreases early in senescence due to a decrease in its stability Lamin B1 silencing leads to the formation of misshapen nuclei and nuclear blebs	Dou et al. (2015), Dreesen, Chojnowski, et al. (2013), Dreesen, Ong, Ong, Chojnowski, and Colman (2013), Freund et al. (2012), Ivanov et al. (2013), Lattanzi et al. (2014), Liu et al. (2011), Petrini et al. (2017), Scaffidi and Misteli (2005), Shimi et al. (2011), Swanson et al. (2013), Taimen et al. (2009)
LAP2	Human HGPS fibroblasts Senescent human fibroblasts Human skin biopsies from old donors	Reduction in LAP2s (LAP2α and LAP2β) levels in HGPS, senescence and normal aging	Progerin expression down‐regulates LAP2α at the transcriptional level LAP2α decline contributes to the progerin‐dependent impaired proliferation in HGPS LAP2α deficiency changes lamin A/C–chromatin interactions toward heterochromatic regions	Dreesen, Chojnowski, et al. (2013), Gesson et al. (2016), Liu et al. (2011), Scaffidi and Misteli (2005), Vidak et al. (2015)
SUN1	Human HGPS fibroblasts Human centenarian fibroblasts *Lmna*ΔE mutant mice	SUN1 is upregulated and accumulates at the NE and Golgi	SUN1 recruitment to the NE is enhanced due to higher affinity for both the progerin and farnesylated prelamin A, in HGPS and during normal aging, respectively Over accumulation of SUN1 arises from reduced protein turnover, rather than increased transcription SUN1 over accumulation at the NE correlates with NE abnormalities observed in HGPS, namely the heterochromatin profile and cellular senescence	Chen et al. (2012), Chen et al. (2014), Haque et al. (2010), Lattanzi et al. (2014)
Emerin	Human aged‐iPSCs Mouse HGPS fibroblasts	Increase of emerin expression Emerin is mislocated around nucleus	—	Petrini et al. (2017), Sola‐Carvajal et al. (2019)
Nesprin‐2	Human aged‐iPSCs Human HGPS fibroblasts	Increase of nesprin‐2 expression in human aged‐iPSCs Decrease of nesprin‐2 at the nuclear rim in human HGPS fibroblasts	—	Petrini et al. (2017); Sola‐Carvajal et al. (2019)
Nup153	Late passage VSMCs Human HGPS fibroblasts *Caenorhabditis elegans*	Nup153 mislocalization: decrease import into the nucleus and incorporation in NPCs Nup153 protein levels decrease	Nup153 mislocalization affects NPC assembly which impairs large cargo transportation to the nucleus and compromises the DNA damage repair	D’Angelo et al. (2009), Cobb et al. (2016), Larrieu et al. (2018)
Nup93	Old rat neurons nuclei *Caenorhabditis elegans*	Depletion of Nup93	Nup93 loss contributes to the age‐related deterioration of the NPC permeability barrier	D’Angelo et al. (2009)
Nup62	Old rat neurons nuclei	Depletion of Nup62	Nup62 loss contributes to the age‐related deterioration of the NPC permeability barrier	D’Angelo et al. (2009)
Tpr	HGPS fibroblasts	TPR mislocalization: decreased import into the nucleus and defective anchorage at NPCs	Disruption of the nuclear anchorage of the Tpr as a consequence of the failure to import Nup153 Tpr‐mediated anchorage of chromatin to the NE is compromised leading to chromatin disorganization and gene expression alterations	Larrieu et al. (2018), Snow, Dar, Dutta, Kehlenbach, and Paschal (2013)

Note

CCFs, cytoplasmic chromatin fragments; HGPS, Hutchinson–Gilford progeria syndrome; INM, inner nuclear membrane; IPSCs, induced pluripotent stem cells; MEFs, mouse embryonic fibroblasts; NE, nuclear envelope; NPC, nuclear pore complex; SADs, senescence‐associated distension of satellites; VSMCs, vascular smooth muscle cells.

Previous studies indicated that long‐lived NPC structure deteriorates with time, thereby increasing nuclear permeability across the lifespan. These authors observed that Nup93 was lost in old and permeable nuclei (D’Angelo, Raices, Panowski, & Hetzer, 2009). Interestingly, Nup93 has previously been functionally associated with the NPC permeability barrier (Galy, Mattaj, & Askjaer, 2003), which explains the correlation between the age‐related loss of this particular Nup and the increased nuclear permeability observed (D’Angelo et al., 2009) (Figure 2 and Table 1). Additionally, in aged permeable nuclei (where Nup93 was lost), there is a decreased signal of FG‐Nups, detected by the mAb414 antibody that recognizes several Nups, including Nup62 (D’Angelo et al., 2009) (Table 1). Of note, Nup93 was shown to bind FG‐Nups located at the central channel, supporting that the NPC diffusion barrier is established by recruiting those Nups to the pore (Alber et al., 2007; Frosst, Guan, Subauste, Hahn, & Gerace, 2002; Grandi et al., 1997). These deteriorated NPCs showed increased permeability and tubulin accumulation within the nucleus (Figure 2). In old cells, oxidative stress increases the pore leakiness, and the protein components released by NPCs can be found carbonylated. This suggests that oxidative damage might be responsible for the damage accumulation in old NPCs with a consequential decline of nuclear barrier selectivity (D’Angelo et al., 2009) (Figure 2). However, a recent study performed in yeast concluded that oxidative damage is unlikely to be a direct cause for the age‐dependent deterioration of nuclear transport. Alternatively, Rempel et al. (2019) proposed that in aged cells, misassembled NPCs accumulate as a result of NPC assembly quality control decline leading to a reduction of permeable and functional NPCs, and a consequent decrease in the transport dynamics (Rempel et al., 2019).

Studies using aged human fibroblasts also showed a decrease of several nuclear transport factors, namely karyopherin a2, CAS, and RanBP1, with a concomitant reduction in the protein nuclear import rate. These results clearly indicate that the import reduction observed with aging will cause alterations in the activity of crucial transport factors (Pujol, Söderqvist, & Radu, 2002) (Figure 2).

Novel and important insights arise from studying premature aging models which are often applicable to physiological aging (Dreesen & Stewart, 2011; Kubben & Misteli, 2017; López‐Otín et al., 2013). Progerin, the mutant form of lamin A, is responsible for the HGPS premature aging syndrome and for changes in the nuclear lamina structure (Table 1). HGPS patient‐derived fibroblasts have the nucleocytoplasmic transport of several factors with important nuclear functions compromised. The expression of progerin inhibits the nuclear localization of Ubc9 (E2‐conjugating enzyme), disturbs the Ran gradient, prevents Tpr import, and reduces levels of histone H3 lysine 9 trimethylation (H3K9me3) (Kelley et al., 2011). Overall, the nuclear import rate is decreased in HGPS patient cells. Recently, using the same HGPS patient‐derived cells, it was shown that the nuclear import protein transportin‐1 (TNPO1) is sequestered by microtubules and mislocated in the cytoplasm, affecting the nuclear localization of its cargo proteins, namely Nup153 and hnRNPA1. Consequently, there is a disturbance of the Ran gradient, nuclear Tpr anchorage and chromatin organization and gene expression deregulation (Cobb et al., 2016; Larrieu et al., 2018) (Figure 2 and Table 1). Notably, Nup153 and Tpr are the Nups that form the basket located at the NPC’s nuclear side. Remarkably, many of the defects caused by the progerin expression were significantly reduced with remodelin, which is a small‐molecule inhibitor of *N*‐acetyltransferase 10 (NAT10) able to reverse the abnormalities induced by altered nuclear lamina (Cobb et al., 2016; Larrieu, Britton, Demir, Rodriguez, & Jackson, 2014; Larrieu et al., 2018). Indeed, in HGPS cellular models, the NAT10 activity and microtubule stability are increased. This led to proposals that NAT10‐mediated hyperacetylation of tubulin is responsible for the higher association of TNPO1 to microtubules observed in those cells. Recently, it was described that, by inhibiting NAT10, remodelin releases the TNPO1 from microtubules sequestration, enabling the TNPO1‐dependent import of Nup153 and hnRNPA1, thereby rescuing NPCs functionality and improving HGPS cellular phenotypes (Larrieu et al., 2018).

García‐Aguirre et al. (2019) recently described an exacerbation of the exportin 1‐driven nuclear protein export in both HGPS and normal aging, thus affecting the nuclear transport. Exportin 1 mediates the nuclear export of cargo proteins bearing a leucine‐rich nuclear export signal, across the NPC via a Ran‐GTP gradient. In HGPS patient‐derived fibroblasts, the enhanced nuclear export is correlated to progerin‐induced exportin 1 overexpression, and its pharmacological inhibition alleviates the progeroid/aging hallmarks (García‐Aguirre et al., 2019).

In cells where oncogene and replicative senescence is induced, the generation of blebs occurs, which subsequently originate NE‐derived vesicles. Essentially, during NE blebbing, NE budding and cytoplasmic chromatin fragment (CCF) formation occurs (Figure 2), resulting in simultaneous exclusion of chromatin and nuclear lamina fragments together. The CCFs are subsequently degraded at lysosomes (Ivanov et al., 2013). Additional studies have indicated that the LC3, which is an autophagy protein located in the nucleus, binds directly to lamin B1. The complex LC3:lamin B1 mediates the lamin B1 degradation upon Ras‐induced oncogenic insults, via nucleocytoplasmic transport and consequent targeting of lamin B1 to the lysosome (Figure 2 and Table 1). More importantly, when autophagy was inhibited or the interaction between LC3:lamin B1 blocked in primary human cells, the activated Ras‐induced lamin B1 loss is prevented and oncogene‐induced senescence is attenuated (Dou et al., 2015). These results clearly indicate the existence of nuclear autophagy (nucleophagy) in mammalian cells, and two types were recently proposed by Luo and colleagues: macronucleophagy and micronuclear autophagy (Luo, Zhao, Song, Cheng, & Zhou, 2016). Briefly, macronucleophagy was described as a common type of nuclear autophagy, where the nuclear materials destined for degradation are encapsulated and subsequently targeted to autophagic degradation. Lamin B1 together with LC3 is responsible for the autophagosome generation. In micronuclear autophagy, upon genotoxicity, local bleb formation occurs which results in the formation of persistent micronuclei (MN). These MN contain damaged chromosomal fragments, enriched in DNA damage and repair biomarkers, namely γH2AFX, which are degraded by autophagy upon fusion with the lysosome (Luo et al., 2016). Overall, these events of NE remodeling in response to NE shedding/blebbing contribute to NE dysfunction through increased nuclear permeability and, among certain conditions, may induce genome instability (Figure 2).

Overall, nuclear permeability as a result of NPC clustering and degradation as well as NE shedding/blebbing might originate NE ruptures (NERs). These could be transient NERs or irreversible ones. NERs have been reported in viral infections, several types of laminopathies, and different types of cancer cells (De Noronha et al., 2001; De Vos et al., 2011; Denais et al., 2016; Raab et al., 2016; Vargas, Hatch, Anderson, & Hetzer, 2012). Moreover, similar irreversible ruptures have been reported in mitotic MN (Hatch, Fischer, Deerinck, & Hetzer, 2013). It is also well accepted that disturbed or weakened nuclear lamina lead to nuclear rupture (Figure 1). In fact, NE regions where lamins are decreased are more predisposed to bleb formation and subsequent rupture. Additionally, a negative correlation between the A‐ and B‐type lamins’ levels and NER incidence has been described (De Vos et al., 2011; Irianto et al., 2016; Robijns et al., 2016; Vargas et al., 2012). Given that it is well accepted that both A‐ and B‐type lamins are altered throughout aging, one can hypothesize that NERs also increase with the aging process. Essentially, cytoskeletal pressure might originate NE blebs at weak regions (e. g. derived from A‐type lamins depletion) culminating in rupture. Consequently, the exchange of macromolecules as well as soluble components and organelles between the nucleus and cytoplasm occur, and in some cases, these changes are permanent. However, the NE breaks could be repaired quickly, and it has been proposed that barrier‐to‐autointegration factor (BAF) is required for this process. Upon a NER, BAF accumulates and recruits INM LEM domain proteins, endosomal sorting complexes required for transport‐III (ESCRT‐III) membrane repair machinery, and membranes to rupture sites restoring the nucleocytoplasmic barrier (Halfmann et al., 2019). After NERs, one of the components of ESCRT‐III targeted to the rupture site is the CHMPB4. Importantly, when CHMPB4 levels are decreased, the time of NER increases considerably (Denais et al., 2016; Raab et al., 2016; Robijns et al., 2016). The consequences of NERs are somehow dramatic since the loss of NE integrity will perturb cellular homeostasis. An example of this perturbation is that upon NERs, an uncoordinated bidirectional exchange of proteins occurs, some of which are transcription regulatory proteins or complexes, namely Oct‐1, cyclin B, and RelA (De Vos et al., 2011). Of note, depending on the extension and the duration of the rupture that, in turn, depends on repair time, these alterations might cause gene regulatory program alterations. Despite the transient shifts of soluble components caused by ruptures, they also originate permanent macromolecular complex translocations, like PML bodies (De Vos et al., 2011; Houben et al., 2013) and intact organelles, such as mitochondria (De Vos et al., 2011; Vargas et al., 2012). PML bodies are stress sensors and DNA‐processing factories that, in normal circumstances, are located at the nucleus. This mislocation may impact proper DNA maintenance. Furthermore, mitochondria translocation upon NER might represent an additional source of ROS, originating DNA damage (Sieprath et al., 2015). Finally, the repair factors reduction from broken sites may induce damage accumulation (Irianto et al., 2017). Potentially, the unregulated DNA damage could generate genome instability. In fact, it has been proposed that NERs are directly correlated with genome integrity. Interphasic DNA exposed to the cytoplasmic environment is more susceptible to DNA damage as evidenced by increased levels of both γ‐H2AX and 53BP1 (Denais et al., 2016; Raab et al., 2016). Overall, when cells face NERs, they mobilize membrane repair machinery to promote DNA damage repair and redistribute mislocated proteins to preserve gene expression and, consequently, genome stability and integrity.

SUN1, a protein member of the LINC complex, is also associated with NPC and is important for the uniform distribution of NPCs across the nuclear surface (Liu et al., 2007). It was shown that SUN1 is overexpressed and accumulates in HGPS patient‐derived fibroblasts, and that its reduction results in the correction of the nuclear defects and cell senescence (Chen et al., 2012, 2014) (Figure 2 and Table 1). SUN1 displays preferential binding for the farnesylated progerin/prelamin A, resulting in its aggregation and accumulation in HGPS cells that ultimately disturb both NE and ER structures (Chen et al., 2014; Liu et al., 2007). SUN1 overexpression was also correlated with alterations on actin‐dependent nuclear movement and centrosome orientation observed in both HGPS fibroblasts and fibroblasts of aged individuals, as a result of imbalanced nucleocytoskeletal connections (Chang et al., 2019). Moreover, SUN1 overexpression or depletion has been shown to cause clustering of NPCs, similar to the HGPS cellular phenotypes. Therefore, it seems that SUN1 may have a crucial role in the nuclear morphological alterations and NPC clustering observed in HGPS (Chen et al., 2014; Liu et al., 2007).

In summary, several lines of evidence suggest the involvement of NPCs in the pathogenesis of aging and age‐associated diseases. Further research on unraveling the molecular mechanisms underlying NPCs and nuclear lamina damages/loss of function during lifespan will significantly contribute to the understanding of NE stress/NE dysfunction.

## 3 CHROMATIN ORGANIZATION

Higher‐order structure of chromatin organization has received increased attention in recent years. Using advanced techniques like next‐generation sequencing, new perceptions regarding DNA territories organization, and intra‐ and interchromosomal interactions regulation were attained (de Wit & de Laat, 2012). Basically, small and active chromosomes (gene‐rich chromosome territories) tend to be located at the center of the nucleus, whereas inactive and heterochromatic regions (gene‐poor chromosome territories) are at the nuclear periphery, pericentromeric bodies, and perinucleolar regions (Lemaître & Bickmore, 2015; Politz, Scalzo, & Groudine, 2016; Saksouk, Simboeck, & Déjardin, 2015). Some genes are positioned at the nuclear periphery in proximity to the NPCs and associated with transcriptional activity in several organisms (Boyle, 2001; Croft et al., 1999).

Overall, in the nucleus, the gene positioning is not random and the inter‐ and intra‐chromosomal interactions are needed for the regulation of a specific locus, but also for other processes, namely DNA repair and replication. The chromatin fiber plasticity may be restricted by the generation of higher‐order structures through INM proteins’ interactions. The chromatin is stably associated with nuclear lamins, and strong evidence exists supporting that the nuclear lamina together with INM components are crucial for chromatin organization and regulation, via modulating and anchoring heterochromatin and chromosomal domains through interactions with chromatin‐associated and transcription factors. Nuclear lamina is important for the maintenance of genome integrity, whereby it is responsible for the recruitment of the machinery for DNA damage response (Cancino et al., 2011; Gonzalo & Kreienkamp, 2015). Additionally, lamins and INM proteins bind directly or indirectly to chromatin via recruitment of intermediate factors. Interestingly, lamins also bind to mitotic chromosomes (Glass et al., 1993). Moreover, the amino acids 396–430 located in the tail of lamins A/C are responsible for the binding in vitro (Taniura, Glass, & Gerace, 1995) and in vivo (Goldberg et al., 1999; Mattout, Goldberg, Tzur, Margalit, & Gruenbaum, 2007; Taniura et al., 1995) to core histones. Lamins also bind to DNA through AT‐rich sequences, named scaffold/matrix regions (S/MARs) both in vitro and in vivo (Guelen et al., 2008; Ludérus et al., 1992; Zhao, Harel, Stuurman, Guedalia, & Gruenbaum, 1996). Furthermore, both A‐ and B‐type lamins bind to chromatin via interaction with INM proteins containing the LEM domain, namely LAP2, emerin, and MAN2. LAP2α and LAP2β bind to BAF, which is a critical chromatin‐lamina associating factor. BAF is also required for emerin and A‐type lamins assembly during NE reassembly at telophase and may mediate their stabilization at interphase (Haraguchi et al., 2001; Samwer et al., 2017). Further, the LBR that specifically binds to B‐type lamins interacts with HP1 (Polioudaki et al., 2001; Ye & Worman, 1996).

Of note, NPCs also physically interact with the genome and play an important role in the chromatin organization and in transcriptional regulation (Buchwalter, Kaneshiro, & Hetzer, 2019; Sood & Brickner, 2014). Nups have affinity for distinct regions of the chromatin, binding to repressed and active genes (Buchwalter et al., 2019; Casolari et al., 2004). In fact, recently, it was described that while Nup107 targets active sites, Nup93 targets silenced regions bound by polycomb group proteins (Gozalo et al., 2020). Overall, the nuclear periphery, particularly the nuclear lamina together with INM proteins, represents a scaffold platform for the organization of chromatin and chromosomal domains, which are crucial for genome integrity maintenance.

Moreover, using advanced techniques, the genomic 3‐D organization was deciphered and, consequently, specific subdomains identified and described. Among these are the lamin‐associated domains (LADs) and the nucleolar‐associated domains (NADs) that associate chromatin with nuclear lamina and the nucleolus, respectively (Guelen et al., 2008; Van Koningsbruggen et al., 2010). In particular, studies of genome‐wide mapping using the powerful DNA adenine methyltransferase identification (DamID) technique (Steensel & Henikoff, 2000) have indicated the existence of 1,100–1,400 LADs, that corresponds to large regions of the genome related to both emerin and type‐B lamins (Guelen et al., 2008). These regions were subsequently characterized as regions of weak expression, also presenting a mark of repressive chromatin, suggesting heterochromatin sequestration at the nuclear periphery. Interestingly, identical LADs were found in cells expressing lamin B1, LBR, and BAF, and the authors proposed that hypothetically they are structured in two distinct complexes: the first composed of lamin B1 and LBR and the second of A‐type lamins and LAP2, emerin, and MAN1 (Solovei et al., 2013). Of note, in the absence of lamins B1 and B2, or BAF, the LADs can be repositioned, while the positioning of NADs is mainly dependent on BAF and lamin A, indicating a dynamic regulation of LADs and NADs by specific components of the nuclear lamina (Kind & van Steensel, 2014; Padeken & Heun, 2014). Not surprisingly lamins’ depletion, due to aging and aging‐associated diseases, alters the chromatin fiber plasticity and the differentiation capacity of embryonic stem cells (ESCs). Remarkably, a study by Amendola & Steensel (2015) showed that mouse ESCs do not require lamins for LAD organization. The authors proposed that the role of lamin in LADs might be dependent on the cell type and that other components of the nuclear lamina might help to organize LADs (Amendola & Steensel, 2015). However, a more recent study reported decondensation or detachment of specific LAD regions from the nuclear periphery in lamin null mouse ESCs, which alters the chromatin domain interactions and transcription (Zheng et al., 2018). Additional studies indicated that lamins are essential for chromatin organization at early developmental stages (Melcer et al., 2012). Further, the dynamics of heterochromatin proteins, like histone H1, is restricted by *LMNA* ectopic expression (Melcer et al., 2012). LBR binds to H3K9me3 through HP1 (Ye & Worman, 1996) and H4K20me3 via its tudor domain (Hirano et al., 2012), and it is enough to sequester heterochromatin at the nuclear periphery. Downregulation of LBR and lamin A induced alterations in chromatin architecture (Polioudaki et al., 2001; Ye & Worman, 1996).

In HGPS patients’ fibroblasts, it was shown that accumulation of progerin in the nuclear lamina causes alterations in the repressive histone mark H3K27me3 distribution, and in the associations between heterochromatin and nuclear lamina, which ultimately results in a global loss of chromatin compartmentalization (McCord et al., 2013). Moreover, progerin expression leads to depletion of nucleoplasmic lamins A/C and LAP2α (Vidak, Kubben, Dechat, & Foisner, 2015). In turn, ectopic expression of LAP2α rescues the proliferation defects observed in HGPS patients’ cells, but in a lamin A/C‐independent manner (Chojnowski et al., 2015; Vidak et al., 2015). Vidak et al. (2015) suggested that LAP2α rescued the proliferation defects by regulating ECM gene expression, whereas Chojnowski et al. (2015) proposed that LAP2α stabilizes the chromatin structure by increasing H3K27me3 levels and preventing the progerin‐associated DNA damage that resulted in premature senescence. Lamin A/C also associates with euchromatin and lamins A/C‐enriched fractions overlap with those found associated with LAP2α (Figure 2 and Table 1). LAP2α deficiency changes lamin A/C interaction with heterochromatin (Gesson et al., 2016).

Lamin B1 is mainly associated with heterochromatin, whose levels are reduced in multiple models of cellular senescence (Dreesen, Chojnowski, et al., 2013; Freund, Laberge, Demaria, & Campisi, 2012; Scaffidi & Misteli, 2005; Taimen et al., 2009; Wang, Ong, Chojnowski, Clavel, & Dreesen, 2017). In turn, some studies showed that lamin B1 depletion causes premature senescence (Shimi et al., 2011) and changes the histone marks distribution with a dramatic relocation of H3K27me3 (Sadaie et al., 2013; Shah et al., 2013), whereas the overexpression increases the proliferation and delays senescence onset (Shimi et al., 2011). However, recent studies showed that lamin B1 reduction has a minimal effect on cell proliferation but renders cells more susceptible to senescence (Dreesen, Chojnowski, et al., 2013), while lamin B1 overexpression induces senescence (Barascu et al., 2012; Dreesen, Chojnowski, et al., 2013). Moreover, lamin B1 knockout mice studies also challenge the concept that lamin B1 has an essential role in proliferation, given that lamin B1 knockout mice develop to term and developed all the internal organs (Kim et al., 2011), and lamin B1 knockout keratinocytes proliferate in vivo normally (Yang et al., 2011). Hence, the role of lamin B1 in cellular senescence remains controversial and the causal relationship between depletion of lamin B1 levels and cellular senescence deserves further investigation.

Senescence‐associated lamin B1 loss is mainly achieved by transcriptional downregulation and inhibition of its mRNA translation via miRNA‐23a (Dreesen, Chojnowski, et al., 2013; Freund et al., 2012; Shimi et al., 2011). Additionally, Dou et al. (2015) showed that lamin B1 levels could be affected by elimination via autophagic degradation in the lysosomes through interaction with LC3 in senescent cells (Dou et al., 2015) (Figure 2). Interestingly, LC3 binds poorly to lamins A/C and B2. There is a perfect overlap between LC3 and lamin B1 binding places that corresponds to LAD domains of the heterochromatin, associating the autophagy process and the dramatic chromatin alterations with senescence (Ivanov et al., 2013). Overall, in HGPS, NE defects are observed and progerin accumulation correlates with alterations in chromatin architecture reorganization and decreased peripheral heterochromatin thickness, decreased H3K9me3 and HP1, and increased H4K20me3 (Figure 2 and Table 1). In normal aging, these alterations are accompanied by lamin B1 downregulation and degradation by autophagy, indicating an important role of NE/NE dysfunction upon epigenetic alterations related to the onset and progression of cellular senescence and aging. Lamin B1 alterations contribute to the appearance of senescence‐associated distention of satellites (SADS) that consist of large scale decondensation of pericentromeric satellites (Swanson, Manning, Zhang, & Lawrence, 2013) (Figure 2 and Table 1).

During the aging process, many types of cells originate areas of condensed chromatin called senescence‐associated heterochromatin foci (SAHF) (Figure 2). Of note, these SAHFs are not formed in all senescent cells and their assembly is highly dependent on the induction of senescence, and SAHFs are normally related to oncogenic and not replicative senescence (Funayama, Saito, Tanobe, & Ishikawa, 2006; Jeanblanc et al., 2012; Narita et al., 2003; Zhang et al., 2005). SAHF are structures well organized, which accumulate both HP1 and H3K9me3. They are composed of several concentric chromatin layers, where H3K9me3 is in the center surrounded by H3K27me3 (Chandra et al., 2012; Chandra & Narita, 2013). SAHF regulate gene expression, particularly of cell cycle arrest‐associated genes, upon oncogene‐induced senescence due to their relocation to repressive regions (Narita et al., 2003; Zhang, Chen, & Adams, 2007) or by inhibiting DNA damage response (Di Micco et al., 2011). The H3K9me3 chromatin‐enriched domains, like pericentrosome region or telomeres, are not SAHF components but are located at their periphery (Narita et al., 2003; Zhang et al., 2005, 2007). Additional heterochromatin components together with HP1 and H3K9me3 contribute to heterochromatin remodeling, namely MacroH2A (histone variant related to gene silencing), histone cell cycle defective homolog A (HIRA), and anti‐silencing function 1A (Robin & Magdinier, 2016).

In HGPS patient cells, a heterochromatin decrease is observed but they do not have SAHFs (Scaffidi & Misteli, 2006; Shumaker et al., 2006). However, additional data have indicated that there are similarities between SAHF‐ and progerin‐induced senescence that correspond to loss of the GC‐poor contacts of LADs before SAHF appearance. The authors proposed a two‐step mechanism for SAHF formation involving the nuclear lamina disruption (Chandra et al., 2015).

In senescent cells, the chromatin remodeling reflects the nuclear architecture alterations observed at the organism level during physiological aging. Alterations in several histone‐modifying enzymes occur through the lifespan (reviewed in López‐Otín et al., 2013; Zane, Sharma, & Misteli, 2014), and among these are sirtuins (SIRTs), a family of NAD‐dependent deacetylases with multiple roles in metabolism, cancer, and aging (reviewed in López‐Otín et al., 2013). It is well accepted that both SIRT1 and SIRT6 are involved with longevity given their functions in genomic stability, metabolic regulation, and chromatin remodeling (Liu & Zhou, 2013) (Figure 2). Interestingly, SIRT1 activity is dependent of lamin A binding, but not on progerin or prelamin A binding. Moreover, SIRT1 is responsible for the autophagy‐mediated deacetylation of H4K16 (Füllgrabe et al., 2013), connecting lamin A expression to the autophagic action. In turn, hMOF is an acetyl transferase that also binds lamin A and not prelamin A. This binding mediates hMOF location at the nuclear periphery, and it has been proposed that hMOF:lamin A complex may have a role in autophagy through association with SIRT1, but this role needs to be further investigated. Hence, it seems that nuclear lamina is implicated in both acetylation and deacetylation of histones. SIRT6 also binds lamin A but not prelamin A, resulting in increased SIRT6 enzymatic activity (Ghosh, Liu, Wang, Hao, & Zhou, 2015). SIRT6 deacetylates several lysines of the histone H3 to aid DNA damage repair. Lamin A is fundamental for SIRT6 recruitment to DNA damage sites and adequate chromatin interaction. SIRT6 expression and activity is decreased in HGPS models, contributing to the alterations in DNA damage repair, chromatin structural organization, and telomere maintenance (Endisha et al., 2015; Ghosh et al., 2015). Additionally, lamin A binds to remodeling complexes, namely polycomb repressive complex (PRC) and nucleosome remodeling and deacetylase (NuRD), establishing a repressive heterochromatin state at the periphery of the nucleus possibly through stabilizing the Rbbp4/7 complex, which promotes adequate chromatin structural organization and function (Cesarini et al., 2015; Pegoraro et al., 2009).

## 4 TELOMERE MAINTENANCE

Telomeres are specialized nucleoproteic complexes that in vertebrates are composed of the hexanucleotide repeat TTAGGG associated with the shelterin protein complex, which in turn contains six proteins: telomeric repeat‐binding factors 1 and 2 (TRF1 and TRF2), repressor/activator protein 1 (RAP1), protection of telomere 1 (POT1), TINT1‐PTOP‐PIP1 (TPP1) and TRF1‐ and TRF2‐interacting nuclear protein 2 (TIN2). The shelterin complex is located at the chromosomes’ end and ensures genome stability by protecting the telomeres from the action of DNA damage repair machinery through generation of a characteristic chromatin loop named T‐Loop. Telomeres shorten with each round of cell division, as a consequence of the nonconservative replicative machinery, and, in fact, telomere erosion is observed in aged human tissues (López‐Otín et al., 2013). Of note, in lower eukaryotes, telomeres localize at the nuclear periphery but, in humans, only part of the telomeres are at periphery. This positioning is determined by the proliferative state and also by the nuclear lamina organization (Arnoult et al., 2010; Chojnowski et al., 2015; Crabbe, Cesare, Kasuboski, Fitzpatrick, & Karlseder, 2012; Gonzalez‐Suarez et al., 2009; Guidi et al., 2015; Ludérus et al., 1996). The factors commanding telomere positioning in humans remain poorly understood; however, there is an intriguing relationship between telomeres and nuclear periphery that is discussed below.

In vertebrates, TRF1 and TRF2 bind to duplex telomeric DNA as homodimers (shelterin complex) to preserve the integrity of telomeres (Wood et al., 2014). Telomere length is negatively regulated by TRF1, which may be positioned at the nuclear periphery by lamin B1 during nuclear reassembly upon mitosis. TFR2 forms a protective telomere T‐loop at chromosome ends and interstitial telomeric sequences. This telomere stabilization is dependent on the interaction with lamin A/C and LAP2α (Chojnowski et al., 2015; Dechat et al., 2004; Ludérus et al., 1996; Wood et al., 2014). Further, the interaction of lamin A/C:TRF2 mediates the organization of chromatin loops at the interstitial telomeres which contain the chromatin. Remarkably, in lamin A/C‐depleted cells these structures might be disturbed (Shumaker et al., 2006). Additionally, telomeres are regulated by lamina‐associated proteins. LAP2α colocalize with telomeres at discrete foci at the nucleoplasm (Chojnowski et al., 2015). At the end of mitosis and during NE reorganization, both LAP2α and BAF stably bind to telomeres at the decondensing chromatin region and then co‐segregate with telomeres at the inner nuclear space (Dechat et al., 2004).

Of note, TRF2 does not bind to progerin and, in cells where lamin A/C is absent and in HGPS patient cells, the telomeres are lost (Wood et al., 2014). Moreover, both LAP2α and lamin A/C are decreased in HGPS models (Chojnowski et al., 2015; Naetar, Ferraioli, & Foisner, 2017; Pekovic et al., 2007; Scaffidi & Misteli, 2006; Vidak et al., 2015) (Figure 2 and Table 1). Progerin expression reduces the levels of LAP2α and might prevent the interaction of TRF2 with lamin A/C and, which consequently leads to telomere attrition through TRF2 deregulation (Figure 2). Furthermore, the expression of a dominant‐negative TRF2 protein induces uncapping of telomeres which is well correlated with increased progerin production (Cao et al., 2011). Moreover, TRF2 expression is reduced in response to DNA damage in models of adult‐onset progeroid syndromes caused by *LMNA* mutations through propagation of DNA damage and p53‐mediated senescence (Shah et al., 2013). The INM protein lamina‐associated polypeptide 1 (LAP1) is also involved in lamins and chromatin positioning, and it was recently associated with telomeres function by TRF2 and RIF1 binding. However, the molecular mechanism through which LAP1 and TRF2 regulate telomere function remains to be elucidated (Serrano, Cruz e Silva, & Rebelo, 2016).

The mouse centrosomes and telomeres showed a peripheral clustering localization, whereas a nuclear interior localization is observed in the case of human telomeres (Weierich et al., 2003). An exception was observed during meiosis, when the LINC complex proteins participate in bouquet formation and in the sequestration of telomeres into a subnuclear area together with the paring of homologous chromosomes (Ding et al., 2007; Lottersberger, Karssemeijer, Dimitrova, & Lange, 2015; Penkner et al., 2009; Pereira, Serrano, Martins, da Cruz e Silva, & Rebelo, 2019).

Another recently described component of the shelterin complex is AKT‐interacting protein (AKTIP), which interacts with TRF1, TRF2, and proliferating cell nuclear antigen (PCNA) (Burla et al., 2015). AKTIP is located at the nuclear periphery and binds to lamin A/C, lamin B, and PCNA to control the replication of telomere and stabilization [100]. In HGPS models, the nuclear periphery location of AKTIP is lost (Figure 2) (Burla et al., 2016). Interestingly, AKTIP decrease induces cell senescence that is well correlated with increasing prelamin A expression and the appearance of nuclear abnormalities (Burla et al., 2015, 2016). These data lead the authors proposing a regulatory loop where nuclear periphery changes originate telomere dysfunction and, consequently, the nuclear periphery is affected by shortened telomeres, leading to NE dysfunction.

## 5 FUTURE PERSPECTIVES

**FIGURE 3:**
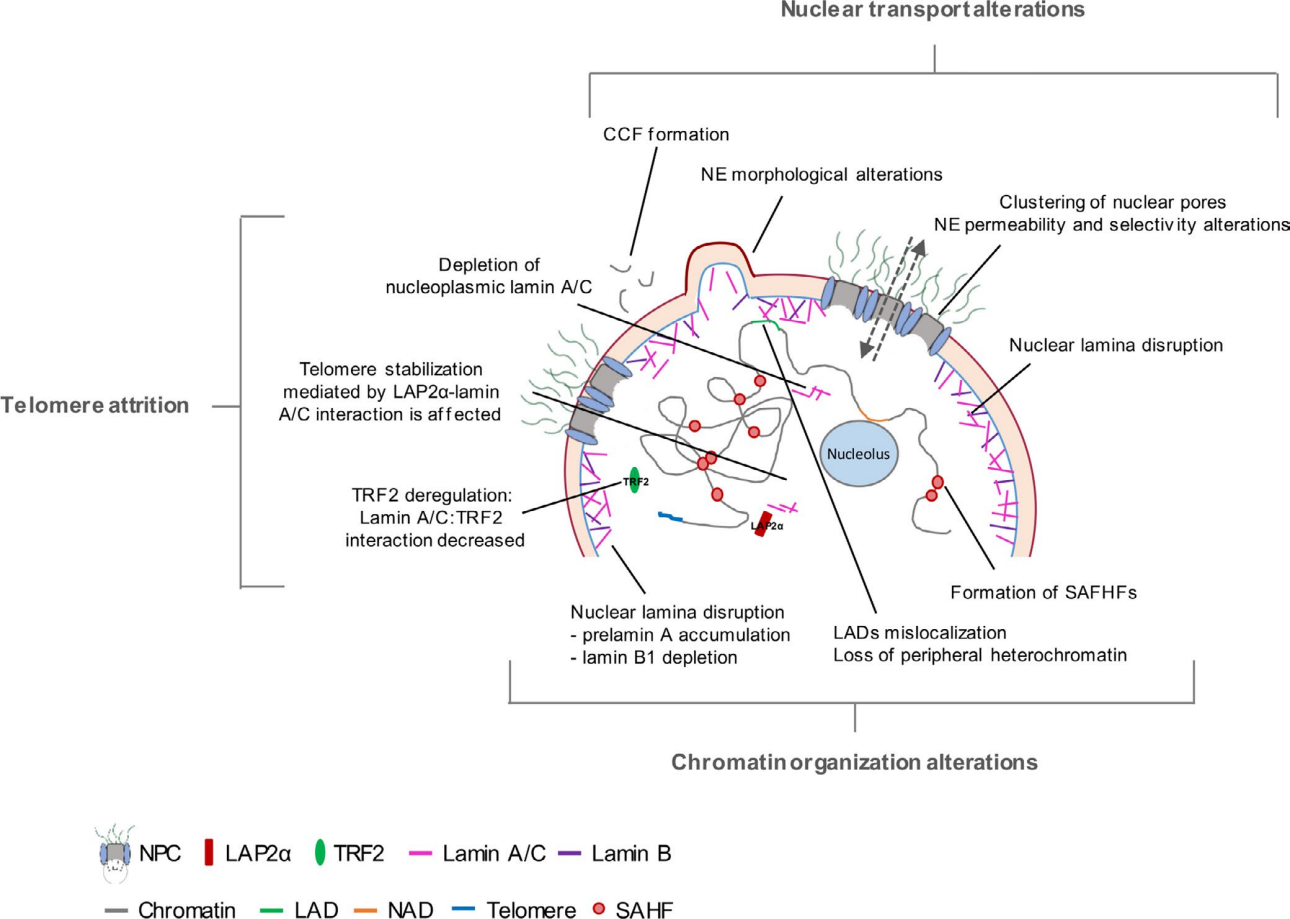
Nuclear transport, chromatin organization and telomere maintenance alterations upon physiological or premature aging. CCF, cytoplasmic chromatin fragment; LADs, lamina‐associated domains; NADS, nucleolar‐associated domains; NPC, nuclear pore complexes; SAHF, senescence‐associated heterochromatin

The increase in life expectancy is one of the highest achievements of humanity; however, aging and age‐related diseases represent a huge challenge, being now considered a health problem affecting millions of people worldwide. Although the underlying cellular and molecular mechanisms of the aging processes remain elusive, the recent years of aging research have provided important achievements. It has been suggested that NE integrity and its dynamic remodeling are pivotal requirements for cellular homeostasis and, consequently, a healthy status. In addition, the huge number of age‐related diseases caused by mutations in nuclear proteins is intriguing and demonstrates that nuclear homeostasis is important for the aging process. Therefore, we could hypothesize that when the NE structure and function and consequently integrity is somehow perturbed, NE dysfunction occurs and could also be a hallmark of aging and of several pathologies, including cancer, viral infection, and laminopathies. The nuclear periphery integrity is achieved by the nuclear lamina (lamins), nuclear lamina‐associated proteins (INM proteins), and NPCs (nucleoporins). Defects in nuclear transport, alterations in chromatin organization and function as well as telomere attrition are correlated with nuclear protein alterations, namely nucleoporins, nuclear transport factors, lamins, INM proteins, chromatin‐associated factors, histones modifications, and sheltering complex proteins, revealing that NE proteins are essential determinants of aging (Figure 3).

The use of adequate aging model systems to study the aging process has limited the understanding of the molecular mechanisms underlying aging. The premature aging disorders (progerias) represent a powerful and unique way to understand physiological aging. In fact, using HGPS patients’ cells it was observed that the nuclear import rate was decreased and that the TNPO1 is sequestered by the microtubules and mislocated at cytoplasm affecting the nuclear localization of the cargo proteins (Nup153 and hnRNPA1). Interestingly, an inhibitor of NAT10 was able to release TNPO1 from microtubules, enabling the Nup153 and hnRNPA1 import, rescuing NPCs functionality and improving HGPS cellular phenotype (Cobb et al., 2016; Kelley et al., 2011; Larrieu et al., 2018). These results are of paramount importance, and hopefully, similar strategies could be applied to physiological aging preventing nuclear transport abnormalities that cause nuclear dysfunction. Nonetheless, more studies should be conducted in order to confirm that the NE dysfunctions observed in progeria are a cause rather than a consequence of HGPS cells undergoing premature senescence.

Moreover, prelamin A and progerin accumulation are features of aging and premature aging diseases, indicating a common mechanism between physiological aging and HGPS. An interesting strategy to prevent the aging phenotype could be related with the maintenance of nuclear lamina integrity, both A‐ and B‐type lamins, given that these proteins together with lamina‐associated proteins are essential for the nuclear transport, chromatin organization and genome integrity, and telomere maintenance (Figure 3).

The hypothesis that nuclear dysfunction is a hallmark of several pathologies, including aging, is emerging. Therefore, unraveling the different molecular mechanisms underlying NE dysfunction or NE stress will significantly impact the understanding of physiological and premature aging.

## CONFLICT OF INTEREST

The authors declare that they have no conflict of interest.

## AUTHOR CONTRIBUTIONS

FM, JS, and SR involved in concept and writing of the manuscript; FM and CDP compiled the table and created the figures. FM, JS, CDP, OABCS, and SR performed analysis of the literature and critical discussion.
